# A comparison of prostate cancer bone metastases on ^18^F-Sodium Fluoride and Prostate Specific Membrane Antigen (^18^F-PSMA) PET/CT: Discordant uptake in the same lesion

**DOI:** 10.18632/oncotarget.26481

**Published:** 2018-12-28

**Authors:** Stephanie A. Harmon, Esther Mena, Joanna H. Shih, Stephen Adler, Yolanda McKinney, Ethan Bergvall, Sherif Mehralivand, Adam G. Sowalsky, Anna Couvillon, Ravi A. Madan, James L. Gulley, Janet Eary, Ronnie C. Mease, Martin G. Pomper, William L. Dahut, Baris Turkbey, Liza Lindenberg, Peter L. Choyke

**Affiliations:** ^1^ Clinical Research Directorate, Frederick National Laboratory for Cancer Research sponsored by the National Cancer Institute, Frederick, MD, USA; ^2^ Molecular Imaging Program, National Cancer Institute, NIH, Bethesda, MD, USA; ^3^ Biometric Research Branch, National Cancer Institute, NIH, Bethesda, MD, USA; ^4^ Laboratory of Genitourinary Cancer Pathogenesis, Center for Cancer Research, National Cancer Institute, NIH, Bethesda, MD, USA; ^5^ Genitourinary Malignancies Branch, National Cancer Institute, NIH, Bethesda, MD, USA; ^6^ Cancer Imaging Program, National Cancer Institute, NIH, Rockville, MD, USA; ^7^ Russell H. Morgan Department of Radiology and Radiological Science, Johns Hopkins University School of Medicine, Baltimore, MD, USA

**Keywords:** prostate cancer, bone metastases, sodium fluoride, prostate specific membrane antigen, spatial analysis

## Abstract

**Purpose:**

Prostate-Specific Membrane Antigen (PSMA) PET/CT has been introduced as a sensitive method for characterizing metastatic prostate cancer. The purpose of this study is to compare the spatial concordance of ^18^F-NaF PET/CT and ^18^F-PSMA-targeted PET/CT within prostate cancer bone metastases.

**Methods:**

Prostate cancer patients with known bone metastases underwent PSMA-targeted PET/CT (^18^F-DCFBC or ^18^F-DCFPyL) and ^18^F-NaF PET/CT. In pelvic and spinal lesions detected by both radiotracers, regions-of-interest (ROIs) derived by various thresholds of uptake intensity were compared for spatial colocalization. Overlap volume was correlated with uptake characteristics and disease status.

**Results:**

The study included 149 lesions in 19 patients. Qualitatively, lesions exhibited a heterogeneous range of spatial concordance between PSMA and NaF uptake from completely matched to completely discordant. Quantitatively, overlap volume decreased as a function of tracer intensity. and disease status, where lesions from patients with castration-sensitive disease showed higher spatial concordance while lesions from patients with castration-resistant disease demonstrated more frequent spatial discordance.

**Conclusion:**

As metastatic prostate cancer progresses from castration-sensitive to castration-resistant, greater discordance is observed between NaF PET and PSMA PET uptake. This may indicate a possible phenotypic shift to tumor growth that is more independent of bone remodeling via osteoblastic formation.

## INTRODUCTION

Most men who develop metastatic prostate cancer in the bone inevitably succumb to the disease [1]. It is thought that once seeding of prostate cancer cells within the bone has occurred and formation of metastasis begins, continued growth and proliferation of cancer cells is dependent on a complex interaction between the cancer cells and the bone microenvironment, leading to a ‘vicious cycle’ of osteoblast recruitment and osteoclastic response within the local area of metastatic invasion [2]. This eventually leads to the osteosclerotic lesion associated with prostate cancer metastases. Radionuclide bone scans have long been regarded as a reliable modality for imaging bone metastases due to their sensitivity in detecting regions of high bone turnover. Several types of bone scans are currently available including ^99m^Tc methylene diphosphonate (MDP) planar bone scans, which are the traditional method, and relatively newer techniques such as ^99m^Tc-MDP SPECT/CT and ^18^F-NaF PET/CT which show superior detection sensitivity [3, 4]. These agents are all taken up in areas of active bone remodeling, specifically new bone formation indicating increased osteoblast activity by osteocytes that are actively laying down bone.

Recently, prostate cancer imaging agents targeting Prostate Specific Membrane Antigen (PSMA), which is highly expressed in prostate cancer cells, have become available. PSMA contains a carboxypeptidase that is thought to cleave glutamate from vitamin B9, possibly activating the phosphoinositide 3-kinase pathway and promoting prostate cancer growth independent of the androgen receptor (AR) pathway [5]. Several PSMA-targeted radiotracers are in clinical trials evaluation, all of which bind to the enzymatic region of PSMA with high affinity and target viable tumor providing a unique biomarker for metastatic disease [6–8].

Previous reports have indicated that there are discrepancies in detection of bone metastases between bone imaging agents and PSMA agents in metastatic prostate cancers [9–14]. These discrepancies are reported *at the lesion level*, i.e. a lesion is seen either on one but not the other. This may depend on disease status (PSMA expressing or not) and the effects of therapy such as suppression of the AR pathway with androgen deprivation therapy (ADT) [12]. However, so far little attention has been paid to spatial colocalization of ^18^F-NaF PET/CT and PSMA PET/CT uptake *within* the same bone lesion. While the microenvironment within prostate cancer bone metastases contains both osteocytes and cancer cells, the presence and degree to which they co-localize has yet to be functionally evaluated across individual lesions. Given the different targeting mechanisms it is feasible uptake distributions may be different within the same lesion, and findings of spatial discordance have potential therapeutic implications for patients with boney prostate metastases. In this study, uptake by both PET/CT agents within individual lesions were carefully mapped by CT co-registration with the purpose to characterize the degree of spatial concordance between ^18^F-NaF PET and ^18^F-PSMA PET uptake.

## RESULTS

Fifty-two patients with metastatic prostate cancer were enrolled across both imaging studies. Nineteen patients met study inclusion criteria with bone metastases in the pelvis or spine co-detected by NaF PET/CT and PSMA PET/CT. Patients were excluded based on soft tissue only disease (N=9), patients with NaF-only bone lesions (N=14), patients with negative scans (N=7), or patients with co-detected lesions not meeting inclusion criteria (N=6). A summary of patient demographics and lesion characteristics are listed in Table 1, with patient-specific characteristics listed in Supplementary Table 1.

**Table 1:** Patient Demographics

Metric	Summary
**N patients**	19
**PSA (ng/mL)**	9.17 (0.27->5000)
**Status**	
CRPC	13
CSPC	6
**Treatment History**	
Untreated	1
Radical Prostatectomy	8
Radiation Therapy	12
Androgen-targeted Therapy	17
Chemotherapy	9
^223^Ra	2
**N lesions detected by both**	
Total^*^	167+
per patient	(1-100+)
**N lesions included in analysis**	
total	149
per patient	5 (1-20)
**Bone regions**	
Ilium	29
Thoracic Spine	41
Lumbar Spine	35
Sacrum	17
Pubis/Ischium	19
Acetabulum	3
Cervical Spine	2
Humeral Head	2
Femoral Head	1

^*^In three patients, true burden estimation was not quantifiable due to N>100 detected lesions by either tracer.

**Figure 1:**
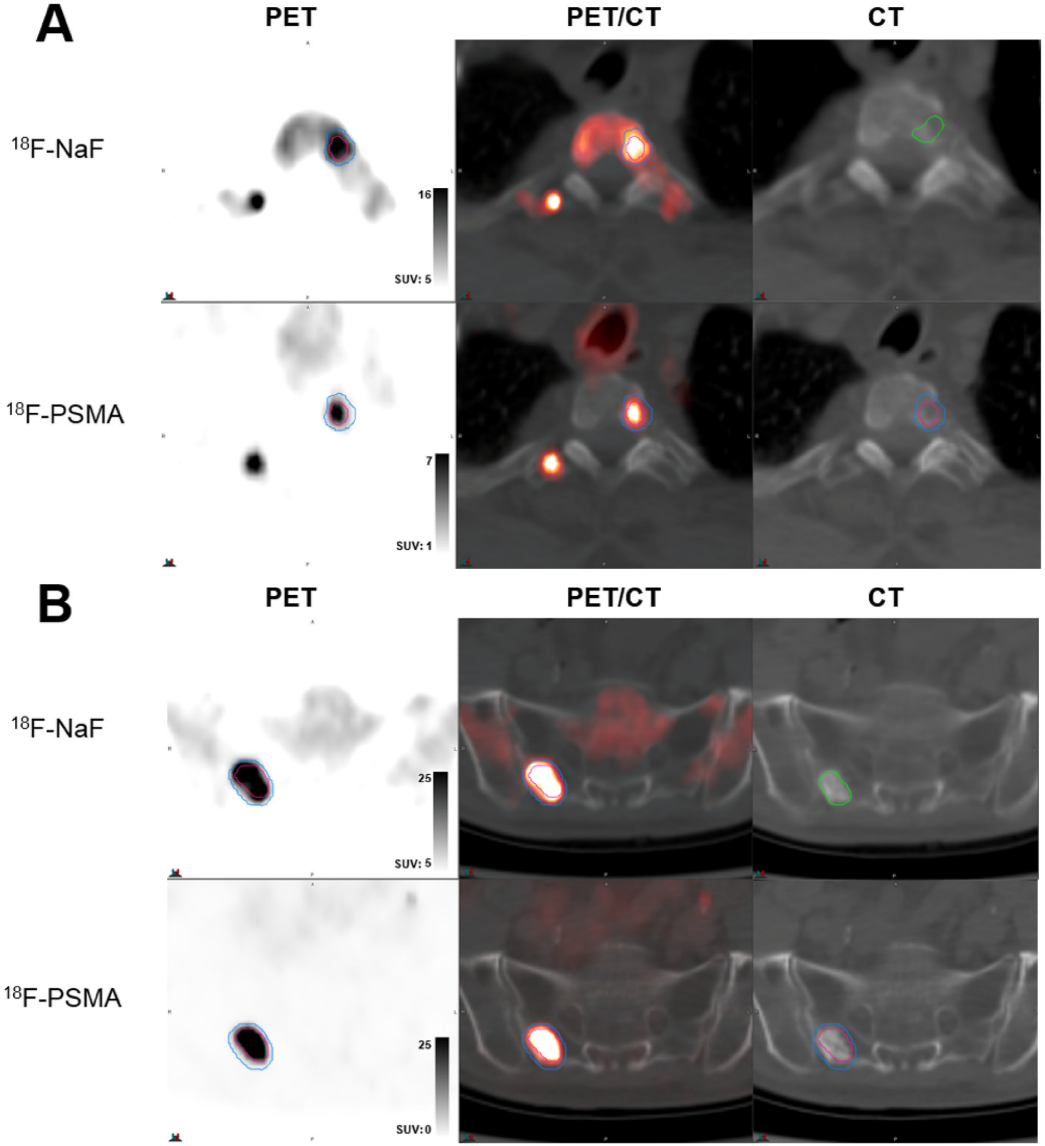
Examples of high volume and spatial uptake concordance **(A)** Lesion in T2 spine of patient newly diganosis with de novo metastatic disease (serum PSA 16 ng/ml) demonstrating 100% volumetric overlap between ^18^F-NaF uptake (top row, blue contour) and ^18^F-PSMA (DCFPyL) (bottom row, red contour) uptake, with CT contour shown in green. **(B)** Lesion in sacrum of castrate-sensitive patient (serum PSA 3.05 ng/ml) demonstrating 88.7% volumetric overlap between ^18^F-NaF uptake (top row, blue contour) and ^18^F-PSMA (DCFPyL) (bottom row, red contour) uptake, with CT contour shown in green. In both (A) and (B), top row demonstrates PET uptake, PET/CT overlay, and low-dose CT from ^18^F-NaF scan and bottom row demonstrates PET uptake, PET/CT overlay, and low-dose CT from ^18^F-PSMA (DCFPyL) scan with registered contour overlay.

**Figure 2:**
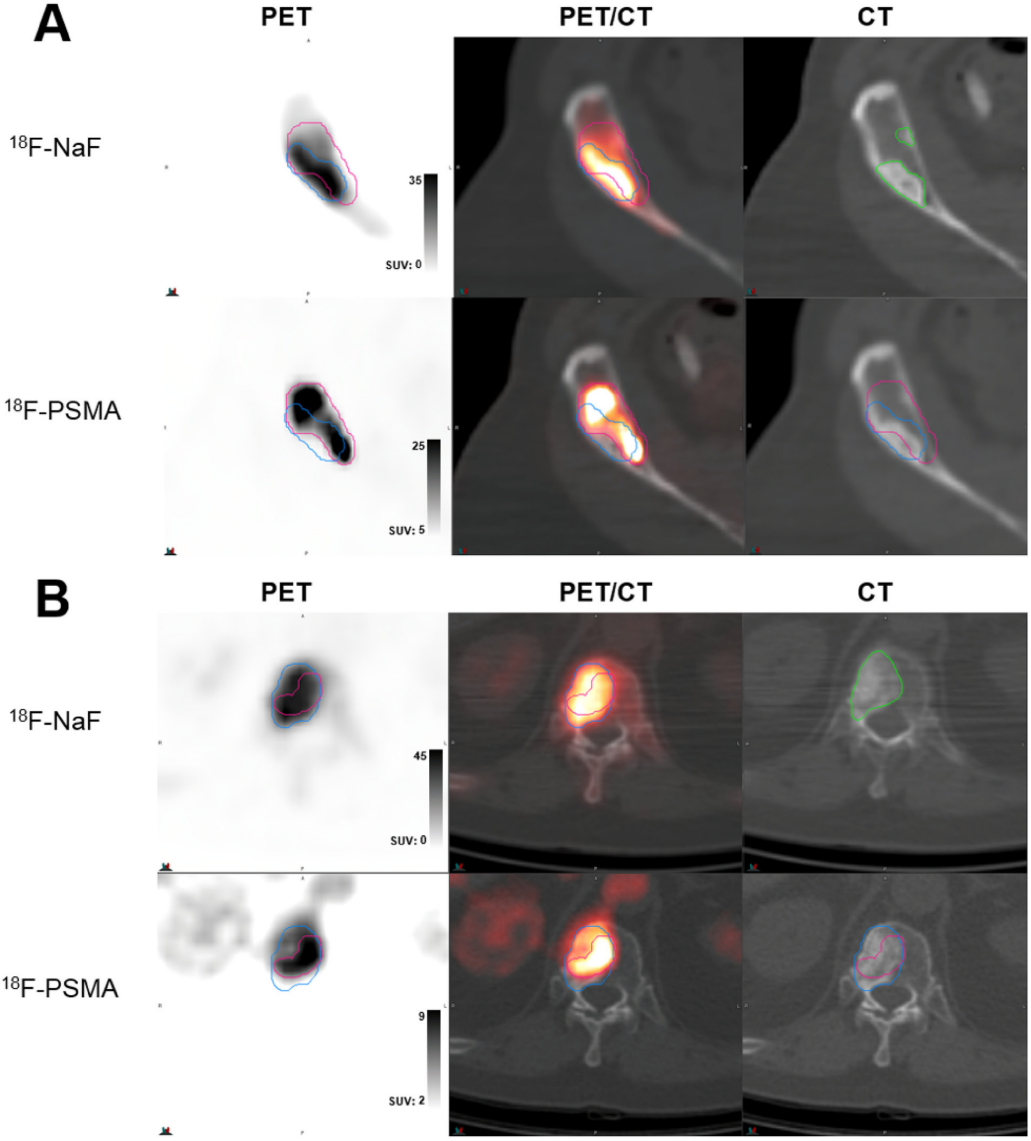
Examples of moderate-to-high volume concordance, with differential localization of highest uptake **(A)** Lesion in anterior iliac crest of castrate-resistant patient (serum PSA 93.3 ng/ml) demonstrating moderate 74.9% volumetric overlap between ^18^F-NaF uptake (top row, blue contour) and ^18^F-PSMA (DCFPyL) (bottom row, red contour) uptake, but mismatching patterns of high uptake within contours and areas of scelrosis on CT (green contour). **(B)** Lesion in T12 spine of castrate-resistant patient (serum PSA 388.1 ng/ml) demonstrating high 95.5% volumetric overlap between ^18^F-NaF uptake (top row, blue contour) and ^18^F-PSMA (DCFBC) (bottom row, red contour) uptake within visible CT lesion (green contour), but differential areas of highest uptake. In both (A) and (B), top row demonstrates PET uptake, PET/CT overlay, and low-dose CT from ^18^F-NaF scan and bottom row demonstrates PET uptake, PET/CT overlay, and low-dose CT from PSMA scans with registered contour overlay.

**Figure 3:**
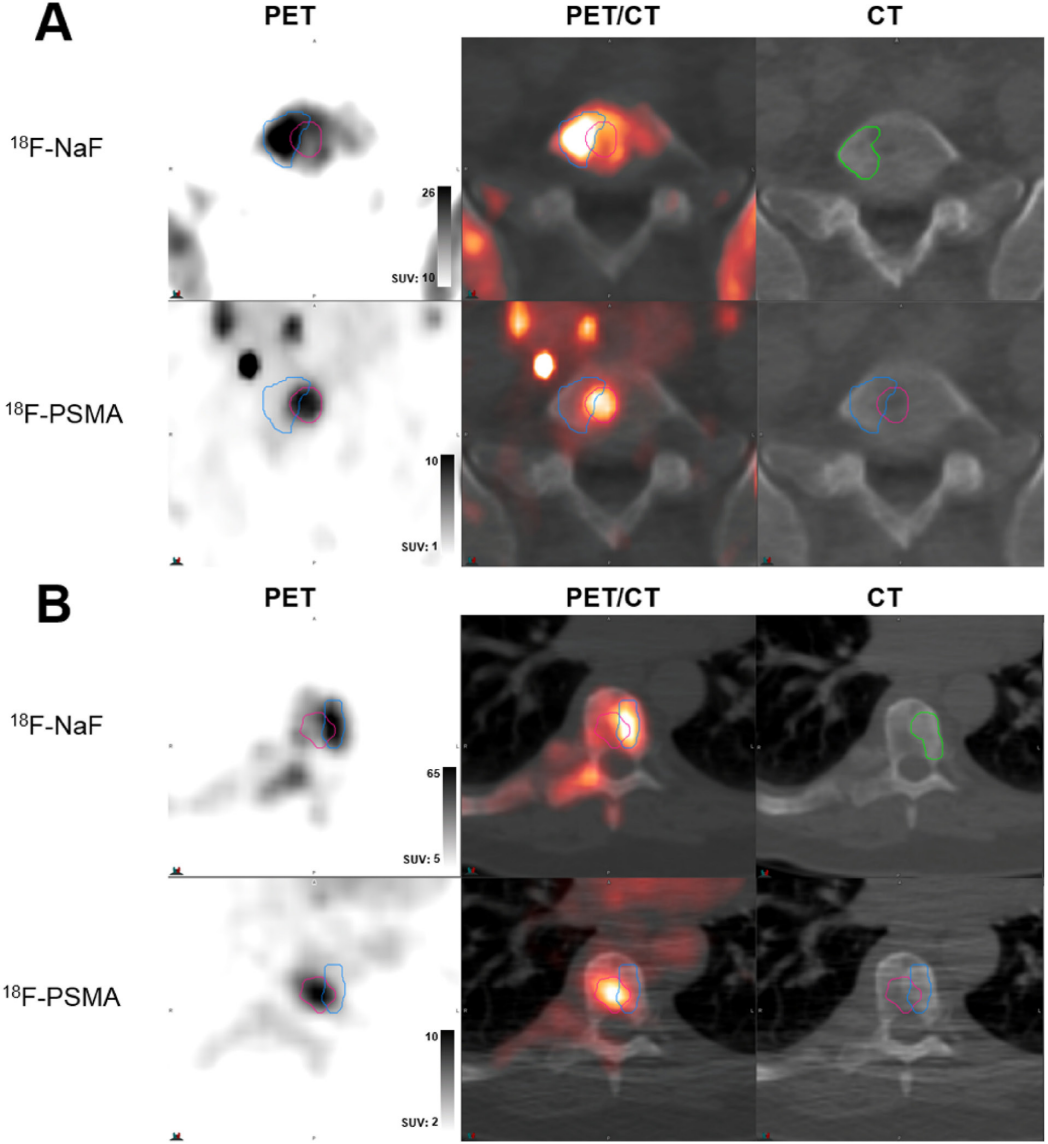
Examples of low volume and spatial uptake concordance **(A)** Lesion in L5 spine of castrate-resistant patient (serum PSA 11.79 ng/ml) demonstrating poor 62.2% volumetric overlap between ^18^F-NaF uptake (top row, blue contour) and ^18^F-PSMA (DCFPyL) (bottom row, red contour) uptake, with marked mismatching patterns of high uptake within contours. **(B)** Lesion in T12 spine of castrate-resistant patient (serum PSA 4379 ng/ml) demonstrating high 30.9% volumetric overlap between ^18^F-NaF uptake (top row, blue contour) and ^18^F-PSMA (DCFBC) (bottom row, red contour) uptake, with substantial mismatch in uptake patterns. In both (A) and (B), top row demonstrates PET uptake, PET/CT overlay, and low-dose CT from ^18^F-NaF scan and bottom row demonstrates PET uptake, PET/CT overlay, and low-dose CT from PSMA scans with registered contour overlay. CT contours shown in green.

224 prostate cancer bone metastases were detected by both NaF and PSMA imaging. 149 of these lesions met inclusion criteria for analysis. Qualitatively, metastases visually exhibited various degrees of matching between the two scan types, as summarized in Figures 1-3. While some lesions demonstrated nearly complete concordance (Figure 1A, 1B) of uptake, others showed regionally-similar distribution manifested as moderate (Figure 2A) or high (Figure 2B) concordance. This further devolved into patterns where partial (Figure 3B) to substantial (Figure 3C) spatial uptake discordance was observed.

Robust CT registration between NaF PET/CT and PSMA PET/CT imaging studies allowed robust registration as was demonstrated by an ICC of 0.926. In the mixed effects model of CT characteristics, Hounsfield Unit (HU) values were shown to vary regionally within voxels categorized by which types of ROIs they were contained within (Table 2). Voxels contained only within PSMA ROIs demonstrated the lowest HU, indicating less sclerosis, while voxels contained only within CT ROIs demonstrated the highest HU (Supplementary Figure 1). Only one lesion in this patient population was characterized by true osteolytic appearance, demonstrating poor spatial colocalization (Supplemental Figure 2).

**Table 2:** Summary of fixed effects estimate from linear mixed effects model of CT (HU) characteristics measured by CT scans acquired during PSMA and NaF PET sessions, using nested random effects model for lesion-based voxel dependencies

Region of overlap	Estimate	SE	p-value
CT exclusive	38.6	1.4	<0.001
NaF-CT only	33.2	1.5	<0.001
PSMA, NaF, and CT	518	reference	
PSMA-CT only	−31.7	2.0	<0.001
NaF exclusive	−179	1.4	<0.001
PSMA-NaF only	−206	1.5	<0.001
PSMA exclusive	−272	1.4	<0.001

Region of overlap was assigned as one of 7 possible categorical memberships for each voxel, using voxels contained within all ROIs (PSMA, NaF, CT) as reference for comparison.

Similar to the qualitative findings, concordance of NaF and PSMA ROI volumes varied substantially across all lesions, with median overlap volume 0.77 (range 0-1). PSMA ROIs showed higher concordance with NaF ROIs compared to CT ROIs (p=0.047), while NaF volumes showed similar spatial overlap with CT (median 0.75, range 0-1) as observed with NaF and PSMA volumes (Supplementary Figure 3). Lesions within the same patient showed varying degrees of spatial discordance and patient-level heterogeneity was observed across metastatic burden (Supplementary Figure 4).

**Figure 4:**
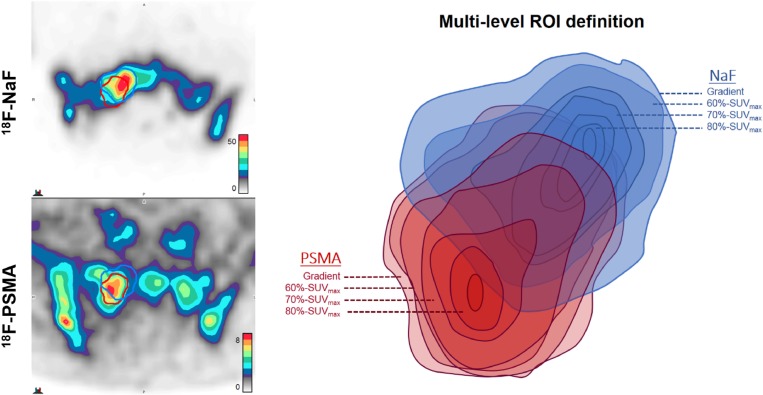
Illustrative example of multi-level segmentation by tracer activity Gradient-based segmentation (outermost level) demonstrates moderate spatial concordance of ROI volumes in right sacral bone lesion of castration-resistant patient (serum PSA 4379 ng/ml) imaged with ^18^F-PSMA (DCFBC) and 18F-NaF. For each radiotracer, additional ROIs are then derived from voxels within gradient-based volumes that fall within 60% of SUV_max_, 70% of SUV_max_, and 80% of SUV_max_. Thus, each ROI is incrementally smaller in volume and higher in uptake relative to SUV_max_ of each tracer within each bone lesion. The example provided demonstrates spatially discordant regions of highest uptake, with decreasing areas of ROI overlap at increasing levels of relative tracer intensity.

**Figure 5:**
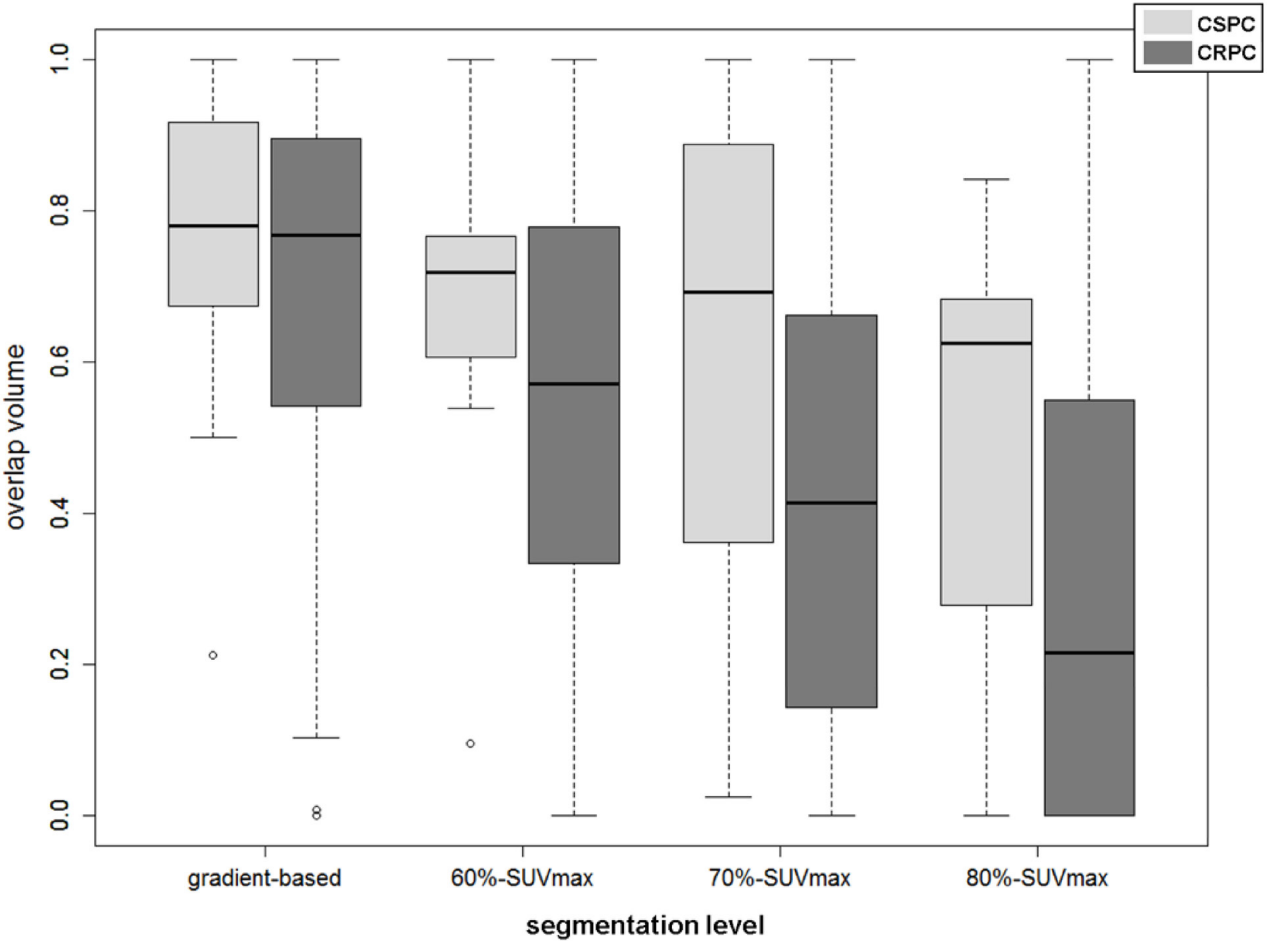
Overlap Volume (OV) vs. segmentation level for CRPC and CSPC patients at various segmentation levels, demonstrating increased spatial discordance at higher levels of tracer activity ranging from gradient-based demonstrating no significant difference (p>0.2), 60%-SUV_max_ ROIs (p=0.15), 70%-SUV_max_ ROIs (p=0.02), and 80%-SUV_max_ ROIs (p=0.0035)

ROI volumes of each bone lesion were further segmented at multiple uptake intensity levels, as described in Figure 4. In this analysis, overlap between NaF and PSMA volumes decreased incrementally at image intensity thresholds of 60%-SUV_max_ (p=0.002), 70%-SUV_max_ (p=0.0005), and 80%-SUV_max_ (p=0.0008). This finding varied according to whether patients were considered to have castration-sensitive (N=6) vs. castration-resistant (N=13) disease, as shown in Figure 5. Metastatic bone lesions in patients with castrate-sensitive disease maintained moderate spatial concordance across all intensity threshold levels (median 62.5% in 80%-SUV_max_ ROIs) while the degree of overlap in lesions within patients with castration-resistant disease decreased significantly at each intensity level (median 21.5% in 80%-SUV_max_ ROIs). Overlap volume in the castration-sensitive group was modestly higher than the castrate-resistant group at 60%-SUV_max_ (p=0.15), becoming significant at 70%-SUV_max_ (p=0.02) and 80%-SUV_max_ (p=0.004). Distance metrics between areas of highest uptake in NaF and PSMA additionally show higher separation (more discordance) in CRPC patients (Table 3).

**Table 3:** Distance measures for CRPC (N = 13) and CSPC (N = 6) patients

Distance Measure	CSPC median (range)	CRPC median (range)	p-value
Distance (mm) between SUV_max_	3.80 (1.94-11.7)	8.91 (0-60.3)	0.001
distance (mm) between 80%-SUV_max_ ROI centroids	2.45 (1.1-5.2)	6.56 (1.0-29.7)	0.002
average pairwise distance (mm) between 80%-SUV_max_ ROIs	4.2 (2.3-10.3)	10.4 (2.8-37.6)	<0.001

Significance was evaluated by permutation test accounting for intra-patient correlations.

Correlation between overlap volume, PET uptake from both radiotracers, and CT characteristics are summarized in Supplementary Table 2. Overlap volume showed weak-to-no correlation to NaF ROI volume (ρ=0.20) and PSMA ROI volume (ρ=0.29), remaining so at all levels of intensity segmentation. Weak-to-moderate inverse correlation was observed for overlap volumes at all image segmentation levels vs. SUV_max_ of NaF, ranging from −0.09 to 0.23, and PSMA, ranging from −0.03 to −0.29 (Supplementary Table 2). A modest, association between HU_mean_ and overlap volume was observed at all PSMA segmentation levels (ranging from ρ=0.31 to 0.36). No correlation was demonstrated for SUV_max_ and HU_mean_ between PET imaging agents and any segmentation level (Supplementary Table 3).

## DISCUSSION

This study demonstrates that many prostate bone metastases are moderately to highly discordant in regard to regions of uptake of ^18^F-NaF PET and ^18^F-PSMA. It is well established that prostate cancer bone metastases induce an osteoblastic bone reaction, visible on bone scans and CT imaging [15]. However, this does not imply perfect concordance between areas of high bone turnover and areas of highest cancer activity. Indeed, it is a common experience that biopsies of sclerotic or bone scan-positive lesions for pathological confirmation of metastatic disease are often negative [16]. While the presence of enhanced bone activity and formation is known to exist in metastatic sites, the spatial concordance with cancer cells has not been functionally evaluated. In this analysis, we report a highly variable spatial concordance of NaF and PSMA PET uptake within the same bone metastases, in which the degree of discordance increases as the disease progresses to castration-resistance.

The disturbance of normal bone homeostasis and active remodeling in sites of skeletal metastatic disease occurs early in the prostate cancer metastatic process and persists throughout the disease, resulting in predominately osteoblastic bone lesions [2, 17]. This knowledge has led to use of the standard MDP bone scan and ^18^F-NaF PET/CT for patient assessment. These imaging agents target areas of increased bone turnover and new bone formation [18]. Spatially, this interaction of prostate cancer cells with the bone microenvironment coincides, such that the region of active bone remodeling usually reflects the region of active cancer [17]. This theory is supported by several findings that tumor burden in a metastatic lesion is regulated by tumor cell interactions with cells within the bone microenvironment, which includes osteoblasts, osteoclasts, and bone stromal cells [19–22]. We find high spatial concordance of osteoblastic activity and cancer-specific PSMA activity occurs more frequently in early (castrate-sensitive) disease.

Lesions in patients with advanced castration-resistant disease [23], show more discordance between regions of osteoblastic activity and PSMA positive tissue. These findings could support the theory that the heterogeneous nature of metastatic prostate cancer, while driven by osteoblastic response initially, eventually evolves to become independent of an osteoblastic response [20], leading to dynamic pathobiologic diversity in end-stage disease [24]. High PSMA PET uptake in areas suspicious for metastasis is considered highly reliable for prostate cancer and discordance with NaF can be interpreted to suggest there is an invasive component not driven by osteoblastic reaction. This parallels bone findings in metastatic breast cancer, where predominately osteolytic mechanisms are suspected to underlay observed discordance between NaF PET/CT and FDG PET/CT [25, 26].

These findings have potential clinical relevance with regard to recently developed therapies for metastatic prostate cancer. ^223^Ra has been approved for treatment of metastatic bone disease [27]. Preliminary studies show spatial correlation between ^18^F-NaF PET/CT uptake and ^223^Ra deposition [28]. Since ^223^Ra is an alpha emitter its region of therapeutic efficacy is narrowly defined (within only a few cell diameters), Use in patients with a high degree of spatial discordance between ^18^F-NaF and ^18^ F-PSMA could potentially result in undertreatment of disease within these lesions.

Different pairs of PSMA PET and bone scan agents have shown differing detection performance of metastatic bone disease in the past [11–13]. Similar detection differences with other PET agents used in metastatic prostate cancer have been reported [29–31]. These findings are likely the result of complex interactions between treatment timing/effect and disease state, underscoring the need for reliable imaging biomarkers throughout the course of disease. In the absence of histological validation for all lesions, inclusion of lesions detected by both tracers allows for phenotypic comparisons within lesions most likely to contain metastatic prostate cancer. Several studies indicate that CT appearance and density correlates to differences in uptake between various radiotracers [31, 32], demonstrating NaF has a higher tumor-to-background ratio in osteosclerotic lesions [13]. Our voxel-based analysis are in general agreement, demonstrating regions defined only by PSMA uptake associate with regions of lower HU.

This study has several limitations. Accurate image segmentation for bone lesions remains an ongoing issue in PET/CT. To account for this fact, multiple image intensity based segmentations were performed in this study to investigate colocalization of tracer uptake without dependency on tumor volume or extent. No partial volume corrections were made, though both cohorts were reconstructed using time-of-flight algorithms, which in combination with point-spread function modeling, may limit the influence of partial volume effects [33]. It is unclear the presence or density of tumor cells and osteoid components within these phenotypic regions. The resolution of PET limits the detection capabilities to the extent that could be achieved in histopathological analysis; however, tissue-based sampling in all metastatic sites is infeasible. Furthermore, the influence of treatment status at the time of imaging on the colocalization of tracers could not be evaluated in this small cohort. A disproportionate number of castration-sensitive patients were in the DCFPyL cohort (6/7); however, similar rates of decreasing overlap volume were observed between imaging cohorts (Supplementary Figure 5). Longitudinal assessment at multiple timepoints by these tracers would be helpful to understand the dynamics of disease activity and tracer uptake over time.

In conclusion, ^18^F-NaF and ^18^F-PSMA PET scans for metastatic prostate cancer can show considerable discordance in regions of increased uptake within a bone metastasis. The relationship between PSMA activity in prostate metastases and bone turnover appears to become weaker in more advanced stages of disease, with metastases occupying portions of bone with no apparent bone turnover or osteoblastic proliferation. In addition to possibly providing insights into the evolution of prostate cancer metastatic spread, these findings have potential implications for radionuclide therapies such as ^223^Ra that depend on localization in areas of increased bone turnover.

## MATERIALS AND METHODS

### Patient population

This study population includes patients with known metastatic prostate cancer undergoing PSMA-targeted PET/CT, using either ^18^F-DCFBC PET/CT imaging (NCT02190279) or ^18^F-DCFPyL PET/CT imaging (NCT03173924), and NaF PET/CT for metastatic disease assessment at a single institution. These studies were approved by the Institutional Review Board and were Health Insurance Portability and Accountability Act compliant. All patients enrolled after written informed consent was obtained. Eligibility required histopathologically confirmed prostate cancer and identifiable metastatic disease on conventional imaging (CT, magnetic resonance imaging or bone scan). All patients received ^18^F-DCFBC PET/CT or ^18^F-DCFPyL PET/CT and ^18^F-NaF PET/CT scans. Clinical demographics, including castration sensitivity/resistance status and prior treatment history were established based on clinical review of patient medical records.

### Image acquisition

Patients imaged between 2014-2016 underwent ^18^F-DCFBC PET/CT and ^18^F-NaF PET/CT scanning (median interval 1 day, range 1-25), as described previously [12]. Patients received ^18^F-DCFBC administered as an IV bolus (median injected dose 8mCi [7.5-8 mCi]) followed by a static whole-body PET/CT performed at 120 minutes post-injection. ^18^F-NaF was commercially obtained (Cardinal Health, Greenbelt, MD). A single, static whole body ^18^F-NaF PET/CT scan was performed 60 minutes after IV bolus (median injected dose 3.5 mCi [3.4-3.6 mCi]) of radiotracer. All imaging was performed on a Philips Gemini TF system (Philips Health Care, Cleveland, OH, USA). Low-dose CT transmission scans were obtained (120 kVp, 60mAs, 0.75 second rotation time, 1.438 pitch, axial slice thickness of 5mm) for attenuation correction and localization. Emission PET images were obtained at 2 minutes/bed position with 22 slices in bed overlap. The PET images were reconstructed using the Gemini TF [34] default reconstruction algorithm (BLOB-OS-TF, a 3D ordered subset iterative TOF reconstruction technique using 3 iterations, 33 subsets, voxel size 4 × 4 × 4 mm^3^).

Patients imaged between 2017-2018 underwent ^18^F-DCFPyL PET/CT and ^18^F-NaF PET/CT scanning (median interval 1 day, range 1-19). Production of^18^F-DCFPyL, a second generation version of ^18^F-DCFBC, has been described previously [35]. Patients received ^18^F-DCFPyL administered as an IV bolus (median injected dose 8.25mCi [8.1-8.4 mCi]) followed by a static whole-body PET/CT performed at 120 minutes post-injection. The ^18^F-NaF imaging procedure was identical for both patient PSMA scan groups (median injected dose 3.46 mCi [3.1-5mCi]). Imaging was performed on a GE Discovery MI DR system (General Electric Medical Systems, Waukesha, WI, USA). Low-dose CT transmission scans were obtained (120 kVp, 2mAs, 0.5 second rotation time, 0.9844 pitch, axial slice thickness of 3.75mm) for attenuation correction and localization. Emission PET images were obtained at 3 minutes/bed position with 22 slices in bed overlap. PET images were reconstructed using Q. Clear method, a Bayesian penalized-likelihood TOF reconstruction algorithm with voxel size 2.73 × 2.73 × 3.27 mm^3^.

^18^F-DCFBC, a first generation agent, suffered from relatively high retention in the blood resulting in slower clearance compared to ^18^F-DCFPyL [6]. However, as ^18^F-DCFPyL and ^18^F-DCFBC agents are chemically related and bind with high affinity to the same PSMA epitope on prostate cancer cells, they are referred to collectively as ^18^F-PSMA scans in this analysis.

### Lesion-based inclusion criteria and image analysis

Standardized Uptake Values (SUVs) were calculated as the ratio of measured activity to injected dose per body weight (kilogram). Image review and analysis was performed using commercial software (MIM v.6.6.10, Cleveland, OH, USA). Lesions determined to be highly suspicious for metastatic disease by consensus of 3 nuclear medicine physicians were considered for analysis. Of highly suspicious bone lesions detected both by NaF PET/CT and PSMA-targeting PET/CT, only those within the pelvis and spine were included in spatial analysis to reduce artifacts introduced from breathing motion (ribs) or patient-positioning motion (extremities, skull). Regions-of-interest (ROI) for each tracer were obtained by semi-automated gradient-based method (PETEdge, MIM). ROIs were further segmented into areas of highest tracer uptake by 60%, 70% and 80% thresholds of lesion-specific SUV_max,_ as described in Figure 4. Regions corresponding to PET-detected lesions were segmented by CT appearance, i.e. encompassing radiographic visibility, by nuclear medicine specialists.

A two-step image registration procedure was completed using low dose CTs. First global skeletal alignment was achieved using rigid alignment. Next, lesion-based alignment was further achieved using local box-based optimization fit to bony regions containing each lesion (Box-based Assisted Alignment, MIM). After registration, spatial concordance of NaF and PSMA ROIs was evaluated by assessing the degree of overlap volume in the area of increased radiotracer uptake, defined as the ratio of overlapping volume to minimum lesion volume. This calculation was repeated for NaF and PSMA overlap with CT. Each voxel was assigned to one of seven possible concordance categories: PSMA exclusive, NaF exclusive, CT exclusive, PSMA and NaF only, PSMA and CT only, NaF and CT only, or all matching (included in all PSMA, NAF, and CT). Distance between regions of highest tracer uptake was measured by, absolute distance from PSMA SUV_max_ and NaF SUV_max_. To avoid potential bias from partial-volume errors, the distance between center-of-mass and average pairwise distance from 80%-SUV_max_ PSMA ROIs and 80%-SUV_max_ NaF ROIs were also calculated.

### Statistical analysis

Voxel-based accuracy of lesion-specific registration was evaluated using the Intraclass Correlation Coefficient (ICC), estimated from a mixed effect model of Hounsfield Units (HU) measurements with nested random effects for patient, lesions, skeletal region and PSMA tracers (DCFBC or DCFPyL) and fixed effect voxel-based concordance category. The significance of fixed effects was determined by the likelihood ratio test. Differences in lesion-based overlap volume as a function of ROI segmentation method was evaluated using paired Wilcoxon signed-rank test using Rosner–Glynne–Lee method to account for intra-patient correlation [36]. Association of overlap volume and distance metrics with disease status (castration-sensitive vs. castration-resistant) was evaluated by permutation test with 2000 permutations at the patient level. Correlation between SUV metrics, PET tumor uptake volume, and CT characteristics were completed using the Spearman correlation coefficient. Standard errors and 95% confidence intervals (CI) were estimated from 2000 bootstrap samples by random sampling on the patient-level. All p-values correspond to two-sided tests, with a p-value <0.05 considered to represent a significant difference between results.

## SUPPLEMENTARY MATERIALS FIGURES AND TABLES



## Abbreviations

PSMA: Prostate Specific Membrane Antigen
NaF: Sodium Flouride
CT: Computed Tomography
SUV: Standardized Uptake Value
HU: Hounsfield Unit
ICC: Intraclass Correlation Coefficient
ROI: Region of interest
MDP: methylene diphosphonate
AR: Androgen Receptor
CRPC: Castrate-Resistant Prostate Cancer
CSPC: Castrate-Sensitive Prostate Cancer
