# Supplementary material for: A comparison of prostate cancer bone metastases on 18F-Sodium Fluoride and Prostate Specific Membrane Antigen (18F-PSMA) PET/CT: Discordant uptake in the same lesion
Source: Oncotarget. 2018 Dec 28;9(102):37676–88. doi: 10.18632/oncotarget.26481 (PMC6340866; doi:10.18632/oncotarget.26481)
Supplement: Supplementary file 1 [file oncotarget-09-37676-s001.pdf]

A comparison of prostate cancer bone metastases on ^{18}F-Sodium Fluoride and Prostate Specific Membrane Antigen (^{18}F-PSMA) PET/CT: Discordant uptake in the same lesion

SUPPLEMENTARY MATERIALS

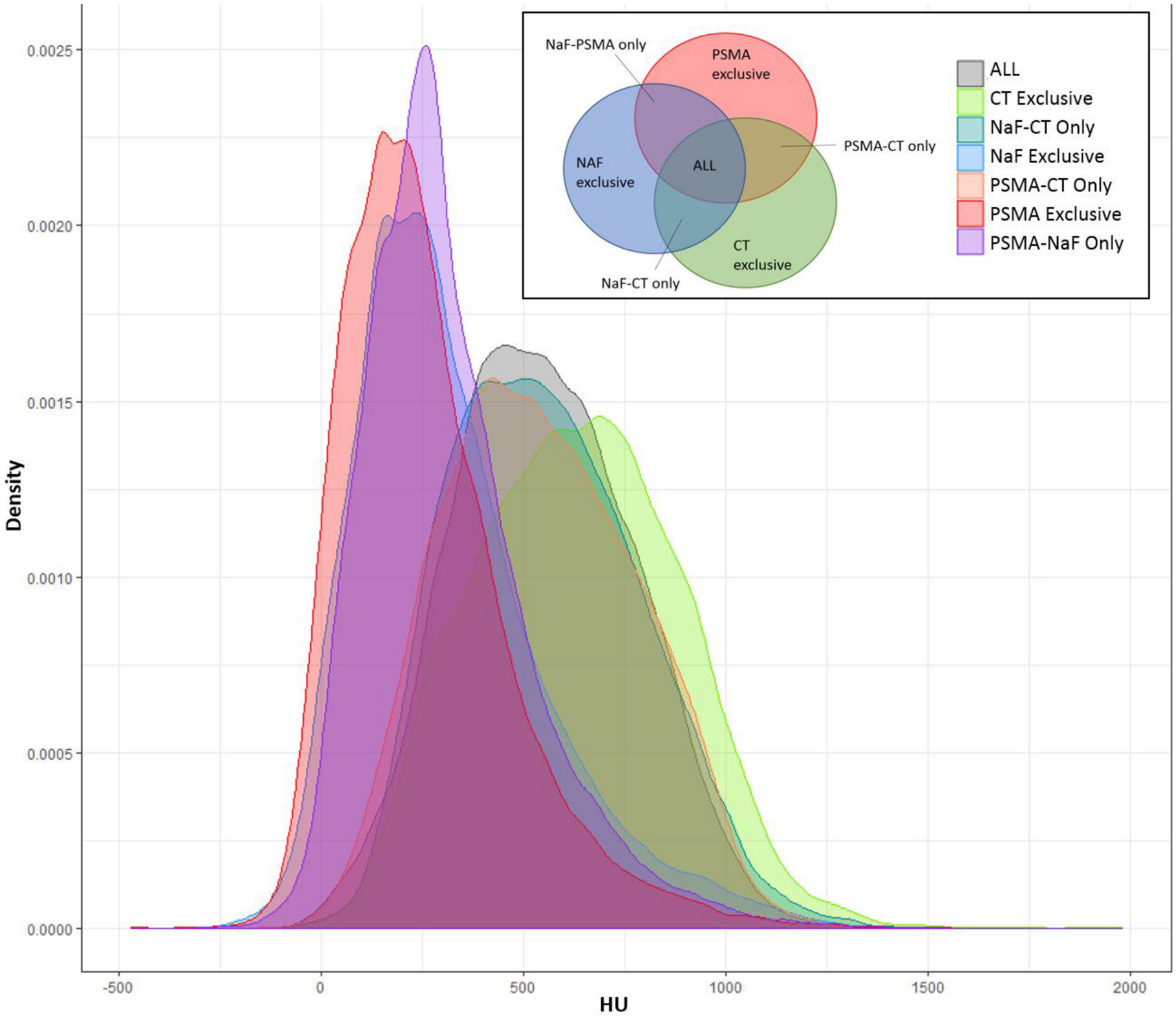

Supplementary Figure 1: Density plot (histogram) of voxel-based CT values (HU) by imaging concordance for both ^{18}F-DCFBC cohort (N=54028 voxels) and ^{18}F-DCFPyL cohort (N=119978 voxels). Voxels contained exclusively within PSMA ROI (**red**) demonstrate lower HU (less sclerotic) compared to voxels contained within all ROIs (**grey**) or voxels exclusively contained within CT ROI (**green**). Similar to PSMA-exclusive regions, voxels contained exclusively in NaF ROIs were less sclerotic than regions overlapping with CT ROIs (**blue**).

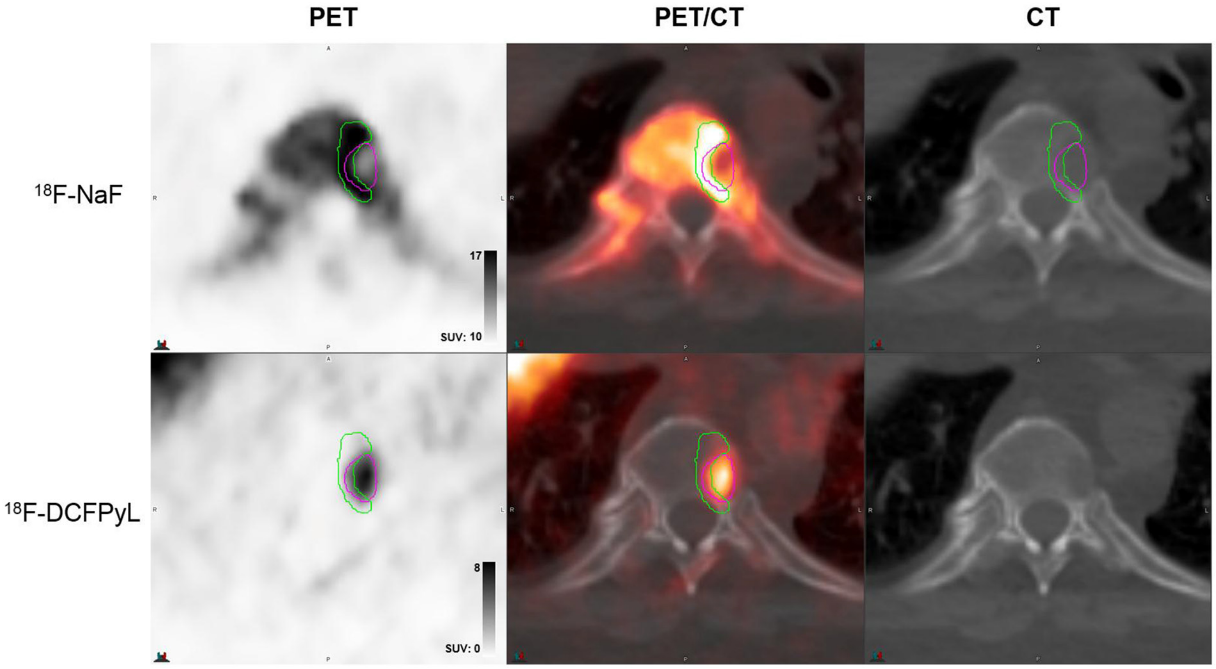

Supplementary Figure 2: Uptake discordance in lytic bone lesion of patient with newly diagnosed prostate cancer (Gleason Grade Group 5 adenocarcinoma with squamous differentiation present) and serum PSA 16 ng/mL. Patient demonstrated 16 metastases detected by both ^{18}F-NaF and ^{18}F-DCFPyL imaging, with 4 meeting study inclusion criteria, of which only one of which was lytic.

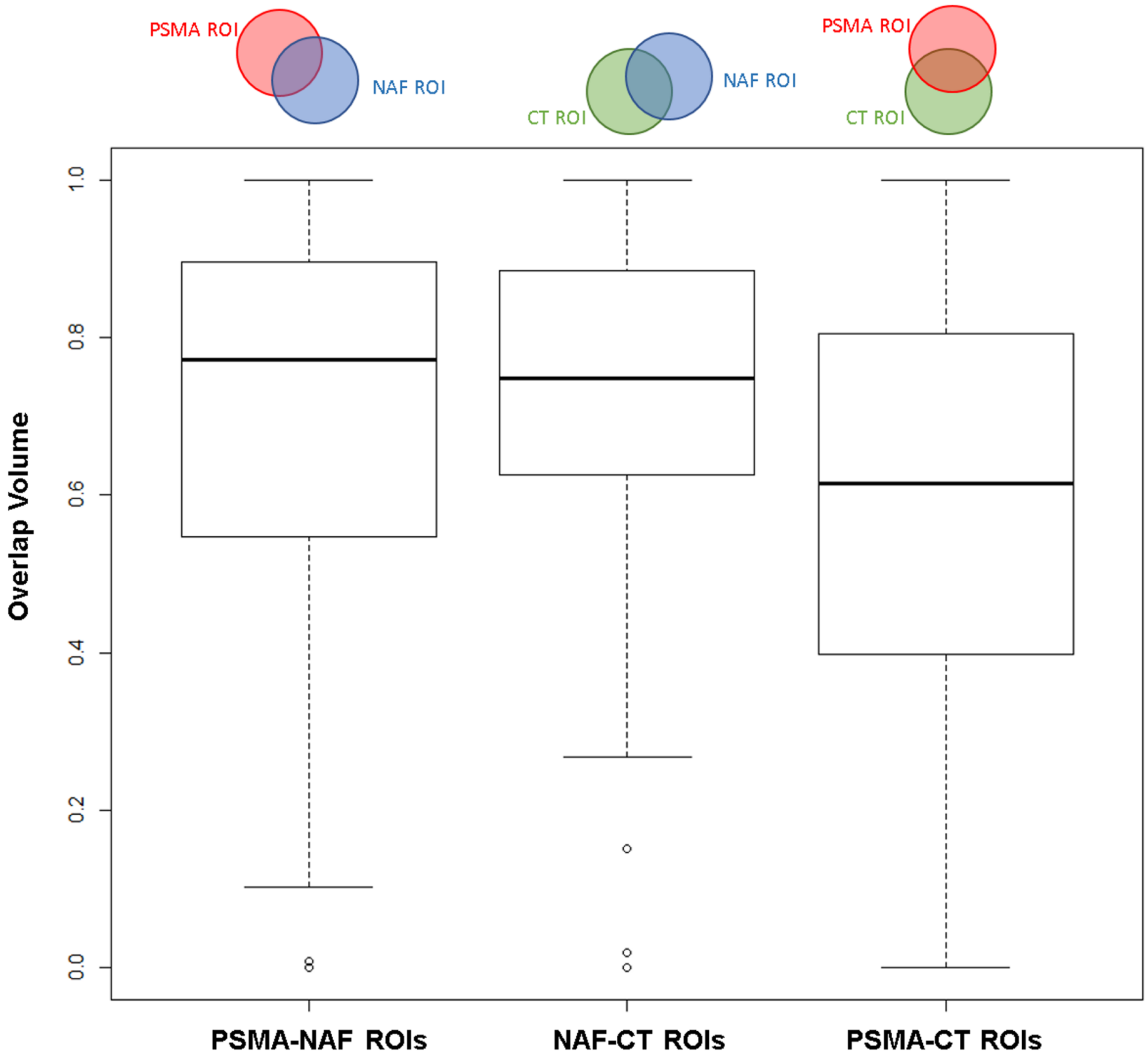

Supplementary Figure 3: Lesion-level Overlap Volume between PSMA-NaF, NaF-CT, and PSMA-CT ROI pairs. Overlap volume was significantly lower in PSMA-CT ROIs compared to PSMA-NaF ROIs (p=0.047).

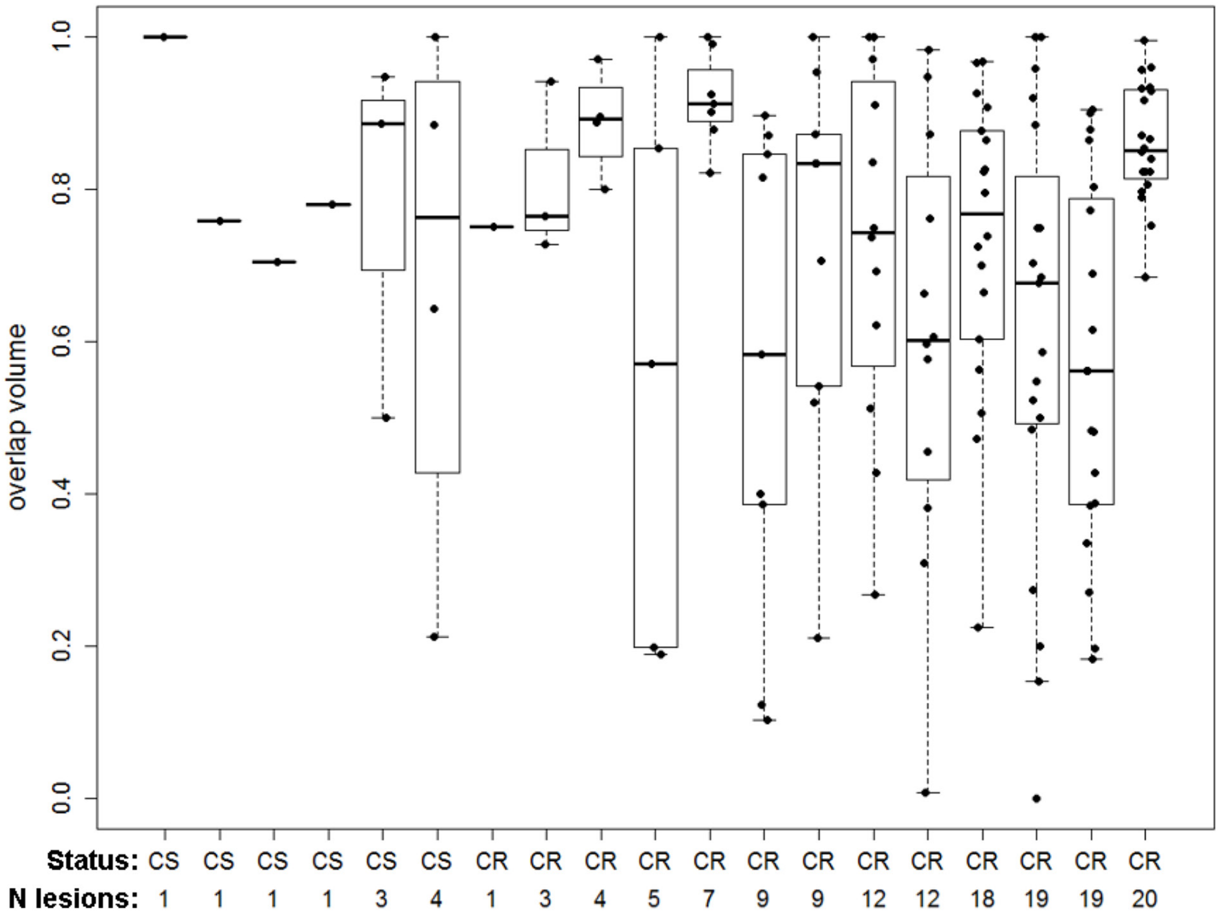

Supplementary Figure 4: Patient-level heterogeneity in overlap volume between NaF and PSMA ROIs with patient disease status (CS = castrate-sensitive, CR = castrate-resistant) and number of lesions included in analysis along x-axis.

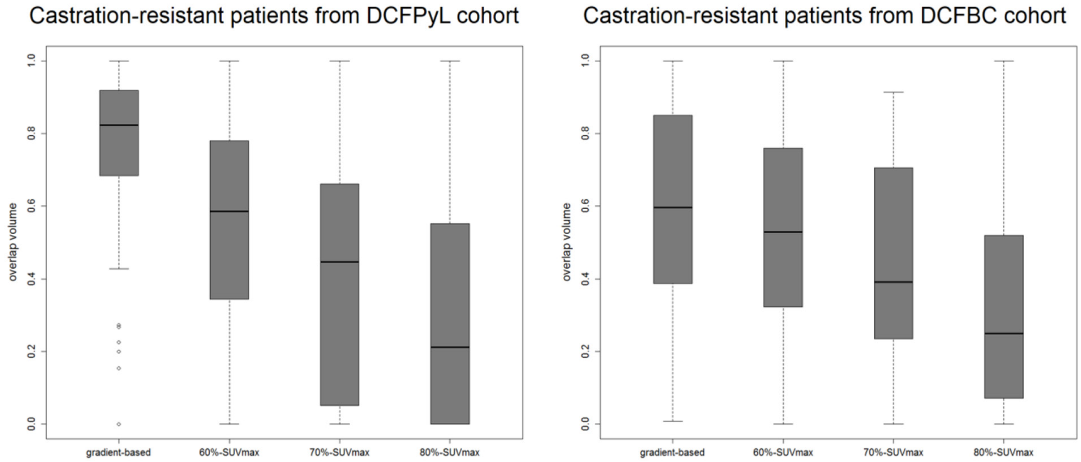

Supplementary Figure 5: Overlap Volume (OV) vs. segmentation level for castration-resistant patients in DCFPyL cohort (left) and DCFBC cohort (right) at various segmentation levels, demonstrating increased spatial discordance at higher levels of tracer activity.

Supplementary Table 1: Summary of patient demographics and treatment history, including number of co-detected bone lesions by NaF PET/CT and PSMA PET/CT and number of lesions meeting inclusion criteria

PSMA agent	PSA	Current therapy	Treatment History	Clinical Status	Number bone lesions detected by NaF and PSMA PET/CT	Number of lesions meeting inclusion criteria
^{18}F-DCFBC	4379	cabazitaxel, enzalutamide	RPx, RT, ADT, docetaxel, abiraterone	CRPC	19	12
^{18}F-DCFBC	0.59	enzalutimide	RT, bicalutimide, leuprolide, degarelix	CSPC	2	1
^{18}F-DCFBC	6.55	bicalutimid	bicalutimide, leuprolide, docetaxel, densoumab	CRPC	19	19
^{18}F-DCFBC	2.08	leuprolide, enzalutimide	RPx, RT, triptorelin, denosumab, degarelix, bicalutimide, vaccine	CRPC	9	5
^{18}F-DCFBC	812.3	goserelin, docetaxel, Cabozantinib	bicalutimide, degarelix, goserelin, provenge, abiraterone, docetaxel, Cabozantinib, denosumab	CRPC	17	9
^{18}F-DCFBC	388.1	leuprolide, abiraterone	RPx, enzalutimide, EBRT, Ra-223, leuprolide, provenge, TAK700	CRPC	15	9
^{18}F-DCFBC	0.27	goserelin	bicalutimide, goserelin, RT	CRPC	1	1
^{18}F-DCFPyL	93.3	leuprolide	casodex, leuprolide, docetaxel, RT, olaparib, enzalutimide	CRPC	>100	19
^{18}F-DCFPyL	>5000	leuprolide	RPx, RT, TAK700, Enzalutimide, Abiraterone, 223Ra, Avelumab, Docetaxel, Cabaitaxel	CRPC	>100	18

PSMA agent	PSA	Current therapy	Treatment History	Clinical Status	Number bone lesions detected by NaF and PSMA PET/CT	Number of lesions meeting inclusion criteria
^{18}F-DCFPyL	5.61	leuprolide, enzalutimide	leuprolide, docetaxel, casodex	CRPC	19	12
^{18}F-DCFPyL	1.73	leuprolide, enzalutimide	hyperthermic therapy, RT, brachytherapy, triptorelin, casodex, enzalutamide	CRPC	8	4
^{18}F-DCFPyL	16	untreated	new diagnosis	CSPC*	16	4
^{18}F-DCFPyL	11.79	none	brachytherapy	CSPC	1	1
^{18}F-DCFPyL	3.05	none	RPx, RT, ADT	CSPC	3	3
^{18}F-DCFPyL	2.61	none	RPx, RT, ADT	CSPC	1	1
^{18}F-DCFPyL	44.45	leuprolide	flutamide, nilutamide, vaccine, docetaxel, thaludomide, prednisone, bevacizumab	CRPC	10	3
^{18}F-DCFPyL	169.6	abiraterone	RPx, degarelix, docetaxel, bicalutimide, enzalutamide	CRPC	27	7
^{18}F-DCFPyL	134.5	cabazitaxel, carboplatin	RPx, RT, ADT, Provenge, Enzalutamide, Abiraterone, Docetaxel, Olaparib	CRPC	>100	20
^{18}F-DCFPyL	4.32	none	RT, brachytherapy, ADT	CSPC	8	1

RPx = radical prostatectomy, RT = radiation therapy, ADT =androgen deprivation therapy (specific therapeutic not specified), Patient marked with (*) indicating untreated de novo metastatic disease, included with CSPC patients for analysis

Supplementary Table 2: Spearman correlation of Overlap Volume (OV) with HU_{mean}, SUV_{max}, and volume within NaF and PSMA ROIs derived from gradient-based, 60%-SUV_{max}, 70%SUV_{max}, and 80%-SUV_{max} segmentations. 95% confidence intervals provided in parentheses

ROI method	PSMA ROI			NaF ROI		
	HU_{mean}	SUV_{max}	volume	HU_{mean}	SUV_{max}	volume
gradient-based	0.31 (0.09, 0.48)	-0.03 (-0.25, 0.21)	0.20 (0.04, 0.30)	0.01 (-0.22, 0.23)	-0.23 (-0.05, 0.46)	0.29 (0.14, 0.43)
60%-SUV_{max}	0.31 (0.10, 0.50)	-0.24 (-0.44, 0.02)	0.01 (-0.24, 0.18)	-0.08 (-0.29, 0.13)	-0.05(-0.27, 0.18)	0.02 (-0.20, 0.18)
70%-SUV_{max}	0.33 (0.11, 0.51)	-0.29 (-0.47, -0.05)	0.06 (-0.25, 0.30)	-0.10 (-0.31, 0.09)	-0.09 (-0.28, 0.10)	0.02 (-0.24, 0.21)
80% SUV_{max}	0.36(0.14, 0.54)	-0.29 (-0.45, -0.08)	0.06 (-0.25, 0.27)	-0.10 (-0.28, 0.10)	-0.09 (-0.25, 0.07)	0.03 (-0.21, 0.21)

Supplementary Table 3: Spearman correlation of HU_{mean} and SUV_{max} within NaF and PSMA ROIs, derived from gradient-based, 60%-SUV_{max}, 70%SUV_{max}, and 80%-SUV_{max} segmentations

ROI method	PSMA ROI	NaF ROI
gradient-based	0.06 (-0.26, 0.31)	0.19 (-0.2, 0.43)
60%-SUV_{max}	-0.09 (-0.36, 0.15)	0.23 (-0.14, 0.46)
70%-SUV_{max}	-0.13 (-0.40, 0.10)	0.20 (-0.15, 0.44)
80% SUV_{max}	-0.15 (-0.39, 0.06)	0.18 (-0.17, 0.43)

95% confidence intervals provided in parentheses.
